# Metformin Impairs Glutamine Metabolism and Autophagy in Tumour Cells

**DOI:** 10.3390/cells8010049

**Published:** 2019-01-14

**Authors:** Serena Saladini, Michele Aventaggiato, Federica Barreca, Emanuela Morgante, Luigi Sansone, Matteo A. Russo, Marco Tafani

**Affiliations:** 1 Department of Experimental Medicine, Sapienza University of Rome, 00161 Rome, Italy; serena.saladini@live.it (S.S.); michele.aventaggiato@uniroma1.it (M.A.); federicabarreca@tiscali.it (F.B.); emanuela.morgante@uniroma1.it (E.M.); 2 Department of Cellular and Molecular Pathology, IRCCS San Raffaele, 00166 Rome, Italy; luigi.sansone@sanraffaele.it (L.S.); matteoantonio.russo@uniroma1.it (M.A.R.); 3 MEBIC Consortium, San Raffaele Rome Open University, 00166 Rome, Italy

**Keywords:** autophagy, cell death, glutaminase, metabolism, molecular rehabilitation.

## Abstract

Metformin has been shown to inhibit glutaminase (GLS) activity and ammonia accumulation thereby reducing the risk of hepatic encephalopathy in type 2 diabetic patients. Since tumour cells are addicted to glutamine and often show an overexpression of glutaminase, we hypothesize that the antitumoral mechanism of metformin could be ascribed to inhibition of GLS and reduction of ammonia and ammonia-induced autophagy. Our results show that, in different tumour cell lines, micromolar doses of metformin prevent cell growth by reducing glutamate, ammonia accumulation, autophagy markers such as MAP1LC3B-II and GABARAP as well as degradation of long-lived proteins. Reduced autophagy is then accompanied by increased BECN1/BCL2 binding and apoptotic cell death. Interestingly, GLS-silenced cells reproduce the effect of metformin treatment showing reduced MAP1LC3B-II and GABARAP as well as ammonia accumulation. Since metformin is used as adjuvant drug to increase the efficacy of cisplatin-based neoadjuvant chemotherapy, we co-treated tumour cells with micromolar doses of metformin in the presence of cisplatin observing a marked reduction of MAP1LC3B-II and an increase of caspase 3 cleavage. In conclusion, our work demonstrates that the anti-tumoral action of metformin is due to the inhibition of glutaminase and autophagy and could be used to improve the efficacy of chemotherapy.

## 1. Introduction

Autophagy is a multi-step recycling process that maintains cell and tissue homeostasis regulated by molecular components encoded by autophagy-related genes (ATGs) [1]. Three principal forms of autophagy have been identified: microautophagy, macroautophagy and chaperone-mediated autophagy [2]. In macroautophagy, hereafter referred to as “autophagy,” cellular substrates or “cargo” are packed into cytosolic vesicles (autophagosomes), which are delivered to lysosomes in order to form double membrane vesicles (autolysosomes). In these structures, the autophagosomal content is digested by lysosomal hydrolases and the products are re-cycled back to cytosol in order to build up new molecules [3]. In mammals, basal autophagy removes damaged macromolecules or refills intermediate metabolites [4].

Autophagy promotes cancer resistance to radiation and chemotherapic treatments [5,6,7] and the abrogation of autophagic machinery renders cervical cancer cells more sensitive to cisplatin [8]. Moreover, a high basal autophagy gives a metabolic advantage to tumours sustaining their elevated energetic demand that arises from rapid proliferation and inadequate blood supply [9].

A link between autophagy and metabolism has been shown by the observation that autophagy can be stimulated also by ammonia, a by-product of glutamine metabolism. Ammonia, in fact, can act both as autocrine and paracrine modulator of autophagy [10]. Ammonia is generated by the mitochondrial glutaminolysis in which glutamine is sequentially deaminated into glutamate and then in α-ketoglutarate entering tricarboxylic acid (TCA) cycle [11]. Notably, it has been shown that under glucose starvation, tumours can survive in vitro using glutamine instead of glucose to maintain cellular ATP production [12]. Tumours show a high rate of glutaminolysis that results in high release of free ammonia. Szeliga et al. [13] observed that glutaminase and glutamate dehydrogenase enzymes, which catalyse glutamine deaminations, are often overexpressed in tumours. At the same time, elevated ammonia favours nitrogen incorporation into amino acids through reductive deamination by glutamate dehydrogenase [14]. In addition, glutaminase inhibition prevents ammonia accumulation and reduces ammonia-induced autophagy, leading to a metabolic crisis that sensitizing tumour cells to death [15].

Interestingly, a recent work has shown that metformin, the most widely prescribed drug for type 2 diabetes (T2D) therapy [16], is independently related to overt hepatic encephalopathy in patients with type 2 diabetes mellitus and high risk of hepatic encephalopathy [17]. Moreover, the same work also demonstrated that, in vitro, metformin inhibits glutaminase activity and ammonia accumulation [17].

This alternative mechanism of metformin could, as well, accompany the reduction of hepatic gluconeogenesis through mitochondrial complex I inhibition. In this case, metformin affects mitochondrial electron transport chain by modifying the AMP:ATP ratio leading to an energetic imbalance [18] that activates protein kinase AMP-activated (PRKAA2) [19]. Once activated, this enzyme, which acts as an intracellular fuel gauge, restores cellular energy balance inhibiting anabolic pathways and promoting glycolysis or fatty acid oxidation [20]. Moreover, activated PRKAA2 is able to inhibit the mechanistic target of rapamycin (MTOR) pathway which regulates cell autophagy [21]. Beyond of this glucose lowering effect, several epidemiological studies have shown that metformin reduces cancer incidence and mortality both in diabetic [22] and in not diabetic subjects [23]. Moreover, diabetic patients with breast cancer cotreated with metformin and neoadjuvant chemotherapy showed a higher pathological complete response than people with T2D on other diabetic treatments [24].

This potential application of metformin in oncology has been evaluated also in in vitro studies performed on a wide range of cancer cells [25,26,27,28,29,30,31]. However, cancer cells used in these studies have been treated for short times (2–3 days) with high doses of metformin (up to 10 mM), far above plasma metformin concentration of people with T2D where the drug achieves a bloody peak at 10–40 μM after 1 h of administration [32].

Starting from these considerations, we explored the effect of metformin on proliferation and autophagy in breast and cervical cancer cell lines where we observed a reduction of cellular replication rate correlated to an inhibition of glutamine metabolism, ammonia production and ammonia-induced autophagy. Moreover, these effects increased when cancer cells were co-incubated with cisplatin.

## 2. Materials and Methods

### 2.1. Cell Culture

Breast cancer cell lines, MCF7 (ATCCHTB-22) and MDA-MB-231 (ATCCHTB-26) and cervical cancer cell line Ca Ski (ATCCCRL-1550) (LGC Standards, Milan, Italy), were grown in RPMI 1640 medium (R0883; Sigma-Aldrich, Milan, Italy). Cervical cancer cell line HeLa was maintained in Dulbecco’s Modified Eagle’s Medium (DMEM, D5648; Sigma-Aldrich). All media were supplemented with 10% Foetal Bovine Serum (Sigma-Aldrich, F9665), 2 mM Glutamine (G7513; Sigma-Aldrich), 100 units/mL penicillin and 0.1 mg/mL streptomycin (P0781; Sigma-Aldrich). Adherent cells were detached by Trypsin-EDTA solution (TA049; Sigma-Aldrich). All cell lines were maintained at 37 °C in a humidified atmosphere of 5% CO_2_ and 95% air.

### 2.2. Treatments Protocols and Antibodies

1,1-Dimethylbiguanide hydrochloride (metformin, D150959; Sigma-Aldrich) was dissolved in distilled water and added to cells at different concentrations (from 5 μM to 10 mM). When used at micromolar doses, metformin was added every day up to 20 days to cells without changing medium. In low-glucose and galactose medium experiments, 2 g/L sodium bicarbonate (E005761; Sigma-Aldrich) and 10 mM galactose (G0705; Sigma-Aldrich) or glucose (G8270; Sigma-Aldrich) were added to RPMI 1640 medium without glucose (R1383; Sigma-Aldrich).

*Bis*-2-(5-phenylacetamido-1,3,4-thiadiazol-2-yl)ethyl sulphide (BPTES, SML0601; Sigma-Aldrich) was dissolved in dimethyl sulfoxide (DMSO, D2438; Sigma-Aldrich) and added to a final concentration of 2 μM. Dimethyl-α-ketoglutarate was synthesized and provided by Prof. Mai (Sapienza University of Rome, Italy) and added to a final concentration of 1 mM. Bafilomycin A1 (B1793; Sigma-Aldrich) was dissolved in DMSO and added to a final concentration of 100 nM. NH_4_Cl (A9434; Sigma-Aldrich) was dissolved in water and added to a final concentration of 20 mM. *cis*-Diamineplatinum (II) dichloride (cisplatin, 479306; Sigma-Aldrich) was dissolved in *N*,*N*-dimethylformamide (DMF, D4551; Sigma-Aldrich) and added to a final concentration of 0.2 μM.

The following primary antibodies were used for western blot analysis: GLS (GTX131263; Gene Tex, Milan, Italy), PHB (NB600-1292; Novus Biologicals, Abingdon, UK), BAX (sc526; Santa Cruz, Heidelberg, Germany), CYCS (Novus Biologicals, NB100-56503), BCL2 (BD, 610538), CASP3 cleaved (9661S; Cell Signalling, Milan, Italy), MAP1LC3B (NB600-1384; Novus Biologicals), GABARAP (PM037; MBL International Corporation, Heidelberg, Germany), BECN1 (9234S; Cell Signalling), SQSTM1 (sc-48402; Santa Cruz Biotechnology), phospho-PRKAA2 (Thr^172^) (PA5-17831; Thermo Scientific, Milan, Italy), PRKAA2 (Thermo Scientific (PA5-36045), ATG5 (MBL, PM050),Phospho-AKT1 (Ser^473^) (9271; Cell Signalling), AKT1 (9272S; Cell Signalling), phospho-RPS6KA1 (Thr^389^) (9234S; Cell Signalling), RPS6KA1 (2708S; Cell Signalling), ACTB (A5316; Sigma-Aldrich), CDK4 (sc260; Santa Cruz). Horseradish peroxidase-linked anti-mouse (NA931V) and anti-rabbit (NA934V) were purchased from GE Healthcare (Chicago, IL, USA).

### 2.3. Generation of GLS-Silenced Cells

MDA-MB-231 cells were stably transfected with a pLKO.1 vector containing a shRNA insert to target human GLS (SHCLND-NM 014905; Sigma-Aldrich). Briefly, 200× 10^3^ cells were plated in 35 mm dishes 24 h before shRNA treatment. The following day the plasmid expressing shRNA GLS (1 μg) was introduced into cells using FuGENE^®^ transfection reagent (E2691; Promega, Milan, Italy) according to manufacturer’s protocol. The day after puromycin dihydrochloride (P9620; Sigma-Aldrich,) was added for selecting stably silenced clones at a final concentration of 1.6 μg/mL.

### 2.4. Viability Assays

Cell viability after prolonged metformin treatment was assessed with different protocols. In the clonogenicity assay cells (2 × 10^3^) were plated in 100 mm dishes to allow clones formation. At the end of metformin incubation, plates were washed twice with a phosphate buffered saline solution (PBS; 79382; Sigma-Aldrich) and fixed with 4% formaldehyde solution in PBS (F8775; Sigma-Aldrich) at room temperature (rt). After 10 min, dishes were washed twice in PBS and stained for 5 min with 0.5% crystal violet (C0775; Sigma-Aldrich). Finally, cells were washed with distilled water and air-dried. The colonies were counted the following day. In trypan blue exclusion assay, cells were seeded on 6-well plates. Following treatments, cells were harvested and stained with 0.4% trypan blue (T8154; Sigma-Aldrich). The cell suspension was applied to a haemocytometer and counted with a phase contrast microscopy (NIKON EclipseTE2000U, Nikon Netherlands, Amsterdam, The Netherlands). Finally, cell viability was checked also by CellTiter96^®^ AQueous Solution Cell Proliferation Assay (G3580; Promega). Cells were seeded in 96-well plates. Following metformin treatments, 20 µL of CellTiter 96^®^ Aqueous Solution was added to 100 µL of culture medium. After 2 h of incubation at 37 °C, absorbance at 490 nm was measured with the GloMax^®^-Multi Detection System (Promega).

### 2.5. Biochemical Assays

Cellular ATP and ADP levels were measured through the ADP/ATP ratio assay kit (MAK135; Sigma-Aldrich) accordingly to manufacturer’s protocol. The amount of glutamate and ammonia produced by cells following metformin treatment was evaluated respectively with Glutamate Assay Kit (MAK004; Sigma-Aldrich) and Ammonia Assay Kit (AA0100; Sigma-Aldrich) as previously reported [14]. Absorbance was read through GloMax^®^-Multi Detection System (Promega). All the experiments were performed in triplicate and data are representative of 3–5 experiments.

### 2.6. Propidium Iodide Staining

To evaluate cell death, cells were analysed by flow cytometry. Cells were seeded into 10 mm dishes and treated with metformin for several days. Cells were next harvested with trypsin-EDTA, washed twice with ice cold PBS and centrifuged at 800× *g* for 5 min at 4 °C. Samples were stained with 50 μg/mL Propidium Iodide (PI, P4864; Sigma-Aldrich) in PBS for 2 h at 4 °C cover light. Fluorescence was read by BD FACS Calibur flow cytometer (Becton Dickinson, Milan, Italy). The sub-G_1_ fraction, which represents the total amount of apoptotic cells, was determined and analysed through CellQuest™ software.

### 2.7. Autophagic Proteolysis Assessment

Click-iT metabolic labelling for proteins (C10428; Thermo Fisher Life Technologies, Milan, Italy) was used to determine autophagic proteolysis of long-lived proteins as previously reported [15]. Cells (70% confluence) were plated on glass coverslips for confocal microscopy and in 96-well plates for fluorometric analysis. The day after, cells were washed twice with warm PBS and then incubated in l-methionine-free medium containing 10% dialyzed foetal bovine serum (26400-036; GIBCO). After 2 h, cells were pulsed for 18 h with 50 μM Click-iT AHA (l-azidohomoalanine), in l-methionine-free medium containing 10% dialyzed foetal bovine serum. At the end of this incubation, cells were washed once with PBS + 3% BSA (A2153; Sigma Aldrich) and cultured for 2 h in complete medium to chase out short-lived proteins. Cells were then treated as indicated in the figure legends. At the end of the treatments, cells were washed twice with PBS, fixed for 10 min with 4% formaldehyde solution in PBS and then washed with 3% albumin from bovine serum (BSA, A9418; Sigma-Aldrich) in PBS. Cells were permeabilized by using 0.2% Triton^®^ X-100 (X100; Sigma-Aldrich) and 0.1 M Tris pH 7.4 (T4661; Sigma-Aldrich) in PBS for 5 min rt. After two washes in 3 % BSA in PBS, alkaline alexafluor 488 (A10267; Thermo Fisher Life Technologies) was added using Click-iT^®^ Reaction Buffer Kit (C10269; Thermo Fisher Life Technologies). The reaction mix was finally removed and samples were washed twice with 3% BSA in PBS before fluorescence detection by LSM 510 confocal microscopy (Zeiss, Milan, Italy) or GloMax^®^-Multi Detection System.

### 2.8. Electron Microscopy

MDA-MB-231 wt and GLS shRNA cells were cultured in 10 mm dishes and treated with metformin 30 μM up to 20 days. In addition, in order to reduce autophagic flux, some samples were treated with NH_4_Cl 10 mM for the last 17 h in the presence or absence of metformin. Cells were washed with warm PBS and fixed with 2% glutaraldehyde (G7651; Sigma-Aldrich) in 0.1 M sodium cacodylate buffer pH 7.3 (C0250; Sigma-Aldrich) at 4 °C overnight. The following day, samples were collected, washed three times with cacodylate buffer and fixed for 2 h rt with 2% osmium tetroxide (75632; Sigma-Aldrich) in the same buffer. After three washes in distilled water, cells were stained for 15 min at room temperature with 1% uranyl acetate. Samples were then incubated at 45 °C with 3% agarose. After solidification, agarose blocks were dehydrated with ascending acetone concentration. Blocks were embedded in Spurr medium and polymerized overnight at 65 °C. Samples were finally cut in 80-nm sections by a Reighert-Jung Ultra cut E ultramicrotome (Leica Microsystems, Wetzlar, Germany) and picked up on copper grids. The tiny pieces were post-stained in uranyl acetate and bismuth subnitrate and observed in a Philips CM-10 TEM (Fei Italia, Milan, Italy) and micrographs on Kodak 4489 sheet films (Sigma-Aldrich).

### 2.9. Lysosomes Labelling

Lysotracker^®^ red DND-99 (L7528; Thermo Fisher Life Technologies) was used to track lysosomes in cells. Briefly, 300 × 10^3^ cells were cultured on coverslips placed inside 35 mm dishes. After 20 days of incubation with 30 μM metformin, cells were washed twice with PBS ad incubated for 30 min in pre-warmed medium containing 50 nM of Lysotracker. Afterwards, fresh medium was replaced and fluorescence was observed by LSM 510 confocal microscopy (Zeiss).

### 2.10. JC-1 Staining

5,5′,6,6′-tetrachloro-1,1′,3,3′-tetrathylbenzimidazolyl-carbocyanine iodide (JC-1) dye was used as indicator of mitochondrial health (T3168; Thermo Fisher Life Technologies). In mitochondria this cationic probe can exist in a monomeric or in an aggregated form depending on mitochondrial membrane potential (ΔΨ_m_). In healthy cells, ΔΨ_m_ is high and JC-1 polymerizes to form J-aggregates which show a red fluorescence emission. On the contrary, in unhealthy or apoptotic cells where mitochondrial integrity is compromised, ΔΨ_m_ assumes a lower value. In this condition, JC-1 remains in a monomeric form showing a green florescence emission. The fluorescence shift from red to green is an indicator of mitochondrial depolarization. Briefly, cells were grown on glass coverslips (for confocal analysis) and in 96-well plate for fluorimeter. At the end of metformin treatment, medium was discarded and 10 μg/mL of JC-1 were added to cells in pre-warmed medium. After 20 min of incubation, cells were washed in PBS and fluorescence was observed by LSM 510 confocal microscopy (Zeiss) or quantified by Epics XL-MCL flow cytometer (Beckman Coulter, Pasadena, CA, USA).

### 2.11. Protein Extraction and Immunoblotting

Cells (2 × 10^6^) for whole cell lysate were centrifuged at 800× *g* for 10 min at 4 °C and pellet were resuspended in 100 μL of a solution containing 50 mM Tris-Cl (93352; Sigma-Aldrich), 250 mM sodium chloride (NaCl, S7653; Sigma-Aldrich), 5 mM ethylenediaminetetraacetic acid (EDTA; E6758; Sigma-Aldrich), 0.1% Triton^®^ X-100 and 0.1 mM Dithiothreitol (DTT, D9163; Sigma-Aldrich) plus 1 mM phenylmethylsulfonyl fluoride (PMSF, 93482; Sigma-Aldrich), Protease inhibitor cocktail (PI; Sigma-Aldrich, P8340), 1 mM sodium orthovanadate (NA_3_VO_4_, S6508; Sigma-Aldrich) and 10 mM sodium fluoride (NaF, 201154; Sigma-Aldrich) (lysis buffer). After 10 min on ice, samples were centrifuged at 14,000× *g* for 10 min at 4 °C and the supernatants were collected. Protein concentration was determined by the Bradford assay (Bio-Rad, Milan, Italy500-0205). Clarified protein lysates (40 μg) were boiled for 5 min, electrophoresed onto denaturatingSDS-PAGE gel and transferred onto a 0.45 μM nitrocellulose membrane (162-0115, Bio-Rad). The blotting membranes were blocked with 5% non-fat dry milk (1706404, Bio-Rad) for 1 h rt and then incubated with primary antibody overnight at 4°C. The follow day, membranes were washed three-times with 0.1% Tween^®^ 20 (P9416; Sigma-Aldrich) in PBS (PBST) for 30 min rt and incubated with the appropriate secondary antibody for 1 h rt. After other 3 washes in PBST, the detection of the relevant protein was assessed by enhanced chemiluminescence (Lite Ablot^®^ TURBO, EMP012001; Euro Clone, Milan, Italy). Densitometric analysis of the bands, relative to ACTB, CDK4 or prohibitin (PHB,) was performed using Image J Software v1.51 (NIH, Bethesda, MD, USA).

### 2.12. Mitochondrial Isolation

Cells (2 × 10^3^) were grown in 100 mm dishes. Following metformin treatment, cells were harvested and centrifuged at 700× *g* at 4 °C for 5 min. Pellet was resuspended on ice in 200 μL of a solution containing 2 mM magnesium chloride (MgCl_2_, M8266; Sigma-Aldrich), 10 mM potassium chloride (KCl, P9333; Sigma-Aldrich) and 10 mM Tris pH 7.4. After 10 min, each samples were mixed with 200 μL of a solution containing 400 mM sucrose (S5390; Sigma-Aldrich), 10 mM Tris pH 7.4, 2 mM EDTA, 2 mM ethylene glycol-*bis*(2-aminoethylether)-*N*, *N*, *N*’,*N*’-tetraacetic acid (EGTA, E3889; Sigma-Aldrich), 2 mM PMSF, 20 mM NaF, 2 mM Na_3_VO_4_ and PI. Cells were broken with 50 Dounce strokes on ice. Homogenates were transferred into 1.5 mL tubes and centrifuged at 900× *g* for 10 min at 4 °C. Pellet were discarded and supernatant fractions were transferred in new 1.5 mL tubes and centrifuged at 17,000× *g* for 30 min at 4 °C. Pellet (mitochondrial fractions) were lysed in 20 μL of lysis buffer and protein concentration determined by the Bradford assay.

### 2.13. Immunoprecipitation

Proteins were extracted as described above. Protein suspensions (500 μg) were pre-cleared with 20 μg of protein A/G PLUS-agarose (sc-2003; Santa Cruz) and kept in slow rotation for 1 h at 4 °C. Samples were centrifuged at 500× *g* for 1 min at 4 °C. Supernatants were collected and agarose pre-cleared resins were discarded. Cleared cell lysates were next incubated with 2 μg of Beclin1 antibody and kept in slow rotation overnight at 4 °C. The following day, 20 μg of protein A/G PLUS-agarose were added to each tube and kept in rotation for 4 h at 4 °C. Samples were then centrifuged at 500× *g* for 5 min at 4 °C. Pellet fractions, containing the protein-antibody complex, were washed 5 times with a solution containing 50 mM Tris pH 7.4, 0.5% Triton^®^ X-100 and 150 mM NaCl and 2 times with 5 mM Tris pH 7.4. At the end of washing, pellet was mixed with 20 μL of Laemmli buffer (NP0007; Invitrogen, Milan, Italy) and heated for 5 min at 95 °C. Samples were electrophoresed on a SDS-polyacrylamide gel and immunoblotted.

### 2.14. Immunofluorescence Microscopy

Cells (2 × 10^3^) were seeded onto coverslips inside 35 mm dishes and incubated for 20 days with metformin. Cells were fixed for 10 min with 4% formaldehyde in PBS, washed twice in PBS and permeabilized for 5 min in 0.1 M Tris pH 7.4 and 0.2% Triton^®^ X-100. After two washes in PBS, samples were blocking for 1 h rt with 0.2 mg/mL BSA and incubated for 2 h rt with anti-Glutaminase C antibody at 1:1000 dilution. Cells were then washed twice with 0.05% Tween^®^ 20 in PBS and incubated for 1 h with the secondary antibody goat anti-rabbit IgG Alexa Fluor^®^ 555 at 1:1000 dilution (A21429; Invitrogen). Finally, samples were washed twice with PBS and mounted using ProLong^®^ Diamond Antifade Mountant (P36961; Thermo Fisher Life Technologies). Fluorescence was observed by LSM 510 confocal microscopy (Zeiss).

### 2.15. Statistical Analysis

The results are expressed as means ± standard deviations (s.d.) and 95% confidence intervals (95% CI) of three independent experiments. Before using parametric tests, the assumption of normality was verified using the Shapiro-Wilk W-test. The Student paired *t*-test was used to determine any significant differences before and after treatment. Significance was set at *p* < 0.05. Statistical software package SPSS v13.0.1. (SPSS Inc., Chicago, IL, USA) was used for all statistical calculations.

## 3. Results

### 3.1. Metformin Inhibits Cancer Cell Proliferation

Metformin has demonstrated an anti-tumoral effect mainly due to the inhibition of mitochondrial function. However, the doses used in the experimental settings are in the order of mM far above the 5–30 μM measured in tissues of patients taking this drug. For this reason we aimed to unravel if and how metformin, used at micromolar concentration, still has anti-tumoral activity.

**Figure 1:**
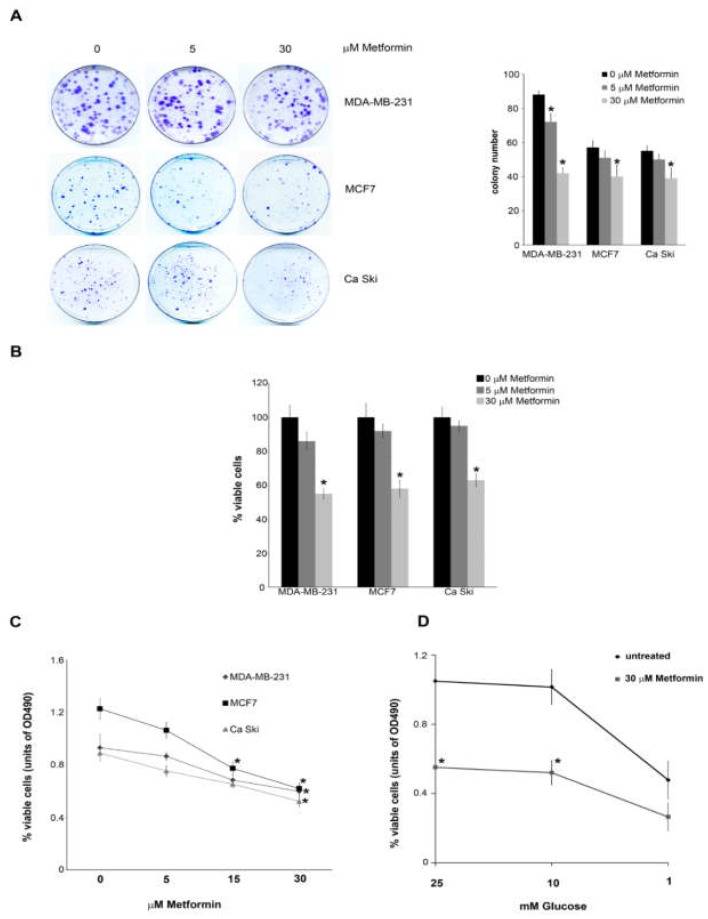
Low doses of Metformin reduce colony formation and cell viability in tumour cell lines; (**A**) MDA-MB-231, MCF7 and Ca Ski cells were seeded at low density (2 × 10^3^) in the presence or absence of micromolar doses of metformin. After 20 days dishes were washed in PBS and cells fixed and stained. Number of colonies were counted the following day and the results graphed in the right side of the figure; (**B**) MDA-MB-231, MCF7 and Ca Ski cells were either left untreated or treated with different doses of Metformin. Cell viability was measured by trypan blue exclusion as indicated under Material and Methods; (**C**) MDA-MB-231, MCF7 and Ca Ski cells were either left untreated or treated with different doses of metformin. cell growth was measured by CellTiter 96^®^ aqueous solution cell proliferation assay as indicated under Material and Methods; (**D**) MDA-MB-231 cells were grown in growth medium containing different glucose concentration in the presence or absence of 30 μM metformin. Cell growth was measured by CellTiter 96^®^ aqueous solution cell proliferation assay as indicated under Material and Methods. All experiments in this figure were repeated three times. * Significantly different from control untreated cells.

To this effect, breast cancer cell lines MDA-MB-231 and MCF7 as well as cervical cancer cell line Ca Ski were treated with 5 and 30 μM metformin for up to 20 days. Clonogenic assay showed a reduction in the number of colonies after metformin treatment (Figure 1A). Moreover, 20 days treatment with 30 μM metformin reduced cell vitality to 55% in MDA-MB-231, 58% in MCF7 and 63% in Ca Ski cells (Figure 1B). Similarly, cell titre assay showed a reduction of cell proliferation in the three cell lines used (Figure 1C). Interestingly, reducing glucose concentration below 10 mM reduced cell proliferation in both untreated and metformin treated MDA-MB-231 cells (Figure 1D).

### 3.2. Metformin Impairs Mitochondrial Function and Induces Cell Death

**Figure 2:**
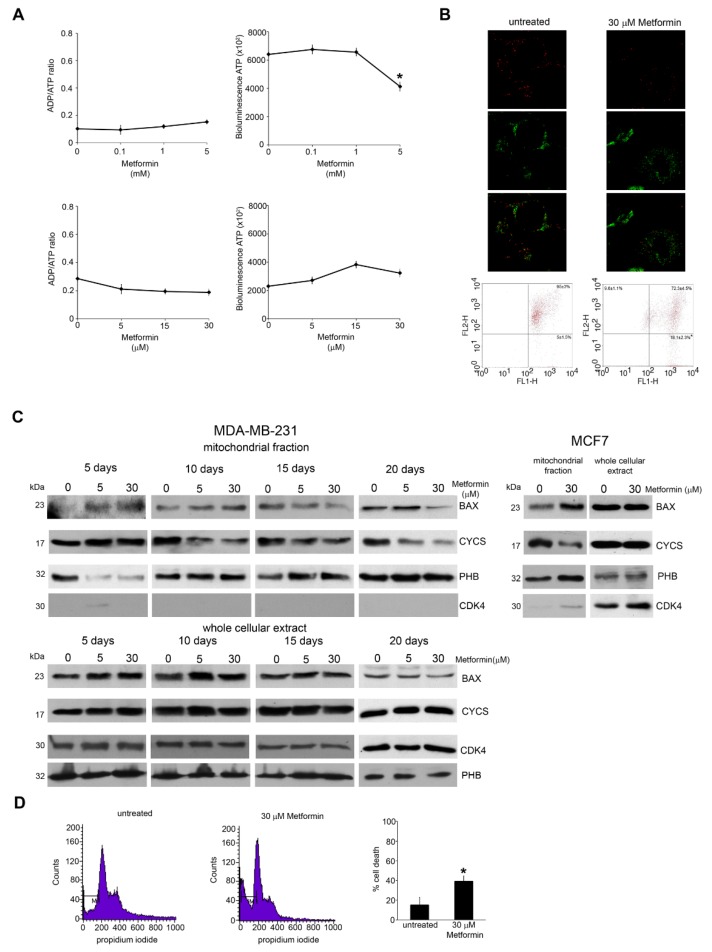
Metformin activates apoptotic cell death; (**A**) MDA-MB-231 cells were plated in 96 well plate and then either left untreated or treated with millimolar (upper graphs) or micromolar (lower graphs) doses of metformin. ADP/ATP ratio was determined as indicated in Material and Methods; (**B**) MDA-MB-231 cells were seeded on glass coverslip and then left untreated or treated with 30 μM metformin. At the end of the treatment, cells were stained with 10 μg/mL JC-1 as indicated under Material and Methods. Mitochondria fluorescence was observed by confocal microscopy. First row: Red fluorescence representing JC1 aggregates. Second row: Green fluorescence representing JC1 monomers. Third row: Merging of the first two rows. Alternatively, cells were treated with 30 μM metformin and stained with JC-1 as above. Red (FL2H) and green (FL1H) fluorescence intensity was quantified by Flow Cytometry; (**C**)upper panel: MDA-MB-231 cells were kept in the presence or absence of metformin for the time indicated and then processed to obtain mitochondrial fractions. BAX and CYCS expression levels were determined by Western blot as indicated under Material and Methods. Densitometric analysis of the gels was performed as indicated under Material and Methods. PHB and CDK4 were used as loading and purity control, respectively. Lower panel: MDA-MB-231 cells were kept in the presence or absence of metformin for the time indicated and then processed to obtain whole cellular extracts. BAX and CYCS expression levels were determined by Western blot as indicated under Material and Methods. Densitometric analysis of the gels was performed as indicated under Material and Methods. CDK4 and PHB were used as loading control. Right panel: MCF-7 cells were kept in the presence or absence of metformin for 20 days and then processed to obtain mitochondrial or whole cellular extracts. BAX and CYCS expression levels were determined by Western blot as indicated under Material and Methods. Densitometric analysis of the gels was performed as indicated under Material and Methods. PHB and CDK4 were used as purity and loading controls; (**D**) MDA-MB-231 cells were kept in the presence or absence of metformin for 20 days. At the end of the treatment cells were harvested and percentage of sub-G_1_ (M1) cells was determined by propidium iodide staining as described in the Material and Methods section. All experiments in this figure were repeated three times. * Significantly different from control untreated cells.

Metformin has been shown to have a direct impact on mitochondrial activity [33]. To test if metformin has different cellular effects if used at milli- or micromolar concentrations, we treated MDA-MB-231 cells with high doses of metformin (mM) for 48 h and low doses (μM) up to 20 days and then we measured ATP levels. As expected, 0.1–5 mM metformin caused a 36% reduction of ATP with an increase of ADP:ATP ratio. Surprisingly, we observed an opposite effect when cells were treated with low doses of metformin for longer times (Figure 2A, lower right). Indeed, in this condition metformin led to a dose dependent increase of intracellular ATP. However, the ADP:ATP ratio did not show any change (Figure 2A, lower left). One possibility is that an energetic unbalance may alter mitochondrial membrane potential (ΔΨ_m_). Therefore, MDA-MB-231 cells were stained with JC-1 [34]. As shown in Figure 2B, 15 days of metformin treatment induced an increase of depolarized mitochondria. In fact, flow cytometry analysis measured a decrease in red fluorescence in 18% of cells treated with metformin compared to the 5% of untreated cells (Figure 2B). Since ΔΨm reduction is critical for apoptosis, we analysed mitochondrial apoptotic markers such as BAX and cytochrome c (CYCS). BAX, a pro-apoptotic member of BCL-2 family, under an apoptotic stimulus oligomerizes to form mitochondrial pores with CYCS release from mitochondria to cytosol, followed by caspase activation and cell death [35,36]. Our results show a mitochondrial accumulation of BAX at 5 and 10 days of treatment and a decrease of mitochondrial CYCS after 10 day of metformin treatment without changes in total amount of BAX or CYCS. Similar results were obtained using MCF-7 cells where, again, 10 days of metformin treatment caused an accumulation of BAX and a release of CYCS from the mitochondria (Figure 2C, right side). The decrease in Bax observed in the mitochondrial fraction after 15 and 20 days in cells treated with 30 μM metformin, could be due to the removal of damaged mitochondria and/or cells after such a long treatment period. In fact, the accumulation of Bax in the mitochondrial membrane is a rather quick process that is followed by mitochondrial damage. Furthermore, damaged mitochondria could then be removed by mitophagy thereby diminishing Bax level. The statistical analysis of Figure 2C is reported in Supplementary Figure S1 showing a statistically significant accumulation of Bax in the mitochondrial after 5 and 10 days of metformin treatment accompanied by a decrease of cytochrome c from 10 to 20 days. The purity of mitochondrial fractions was assessed by using PHB as positive and CDK4 as negative controls as shown in Figure 2C.

Finally, we measured the percentage of MDA-MB-231 cells with sub-G_1_ DNA content after 15 days of 30 µM metformin treatment. Figure 2D shows an increase of cell death from 10% of control untreated cells to 40% of metformin treated cells.

### 3.3. Metformin Inhibits Glutaminase Activity

**Figure 3:**
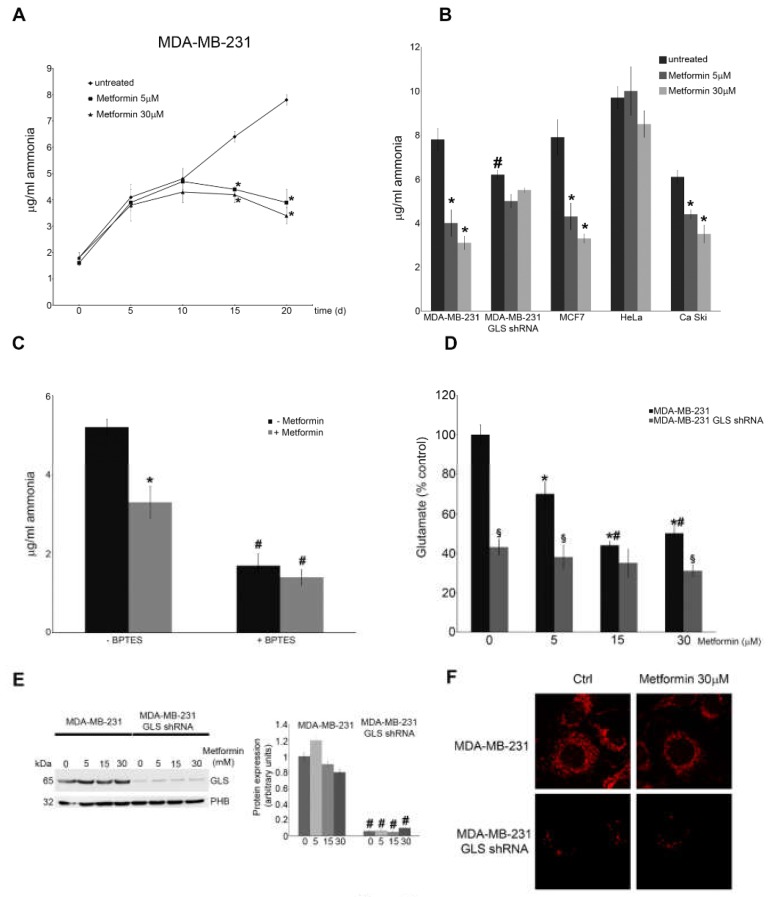
Metformin reduces ammonia and glutamate accumulation by inhibiting glutaminase; (**A**) MDA-MB-231 cells were either left untreated or treated with 5 or 30 μM metformin for the time indicated. Ammonia level in the culture medium was measured as indicated in the Material and Methods section; (**B**) MDA-MB-231, MDA-MB-231 GLS shRNA, MCF7, HeLa and Ca Ski cells were either left untreated or treated with 5 or 30 μM metformin for 20 days. Ammonia level in the culture medium was measured as indicated in the Material and Methods section. ^#^ Significantly different from untreated MDA-MB-231 wt cells; (**C**) MDA-MB-231 cells were either left untreated or treated with 30 μM metformin in the presence or absence of GLS inhibitor 2mM BPTES. Ammonia level in the culture medium was measured as indicated in the Material and Methods section. ^#^ Significantly different from untreated wt MDA-MB-231 cells; (**D**) MDA-MB-231 and MDA-MB-231 GLS shRNA cells were either left untreated or treated with metformin for 20 days. Glutamate level in the culture medium was measured as indicated in the Material and Methods section. ^#^ Significantly different from 5 μM treatment. ^§^ Significantly different from the corresponding treatment in MDA-MB-231 wt cells; (**E**) MDA-MB-231 and MDA-MB-231 GLS shRNA cells were either left untreated or treated with Metformin for 20 days. At the end of the treatment cells were harvested to obtain mitochondria and GLS expression measured by Western blot. Data are representative of at least three separate experiments. Densitometric analysis of the gels was performed as described under Materials and Methods. PHB was used as loading control. ^#^ Significantly different from similar treatment in MDA-MB-231 wt cells; (**F**) MDA-MB-231 and MDA-MB-231 GLS shRNA cells were either left untreated or treated with 30 μM metformin for 20 days. At the end of the treatment cells were fixed and GLS expression determined by immunofluorescence. Data are representative of at least three separate experiments. GLS in red. All experiments in this figure were repeated three times. * Significantly different from control untreated cells.

Next, we measured the amount of ammonia released by MDA-MB-231 cells. As shown in Figure 3A, differences in ammonia release started after 15 days of metformin treatment and became more sustained after 20 days. Figure 3B shows that metformin strongly reduced ammonia levels in a dose-dependent manner also in MCF7 and Ca Ski cells but not in HeLa cells. Indeed, Xiao et al. [37] observed that metformin reduces proliferation of Ca Ski and Me180 but not of HeLa cells. These results suggest that metformin can, directly or indirectly, alter ammonia production in breast and cervical cancer cells. Since ammonia is generated not only by glutamine deamination but also from aminoacidic catabolism [38], we used MDA-MB-231 GLS shRNA to demonstrate that ammonia reduction depends mostly on GLS. We observed that 30 μM metformin inhibited ammonia release in wild type MDA-MB-231 cells but not in MDA-MB-231 GLS shRNA (Figure 3B). Moreover, the basal level of ammonia in untreated MDA-MB-231 GLS shRNA cells was lower than in wild type (Figure 3B). Similar results were obtained in MDA-MB-231 cells co-treated for 15 days with metformin and BPTES, a potent and selective GLS inhibitor (Figure 3C). To further evaluate the effect of metformin on GLS activity, we analysed l-glutamate concentration. Our results showed that prolonged metformin treatments reduced l-glutamate accumulation in MDA-MB-231 cells, an effect that was not observed in GLS shRNA cells (Figure 3D). Finally, we did not observe any difference in GLS expression between control and metformin treated cells (Figure 3E). There results were confirmed by immunofluorescence assay (Figure 3F).

### 3.4. Metformin alters Autophagic Flux

**Figure 4:**
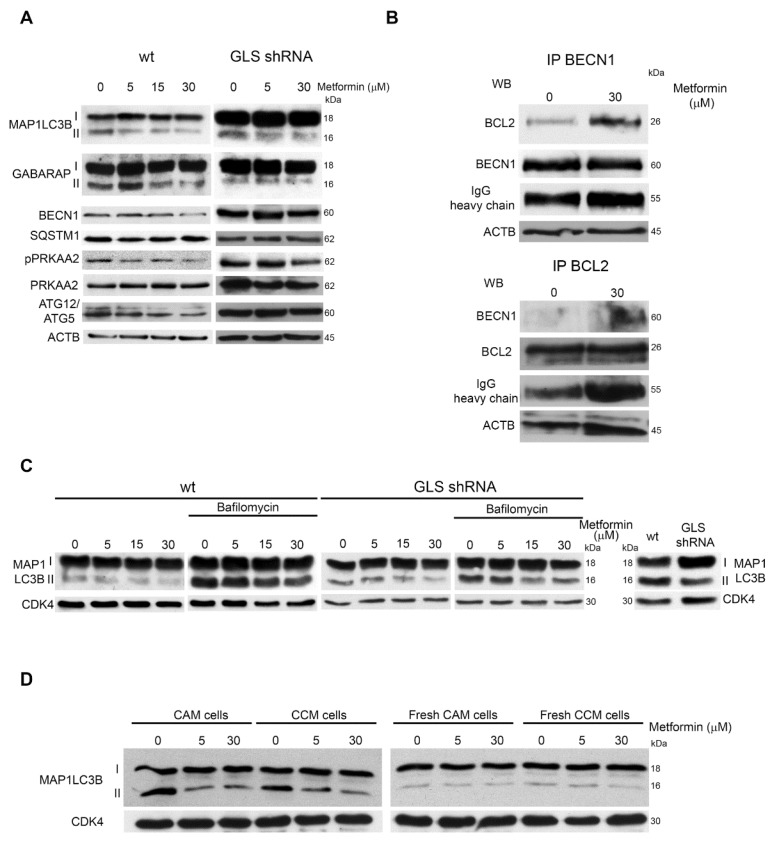
Metformin alters autophagy response; (**A**) MDA-MB-231 and MDA-MB-231 GLS shRNA cells were either left untreated or treated with Metformin for 20 days. At the end of the treatment, cells were processed to obtain whole cellular extracts. Expression level of autophagy markers MAP1LC3B, GABARAP, BECN1, SQSTM1, pPRKAA2, PRKAA2 and ATG12/ATG5 was determined by Western blot. ACTB was used as loading control; (**B**) MDA-MB-231 cells were either left untreated or treated with 30 μM metformin for 20 days. At the end of the treatment, cells were processed. Cellular extracts were immunoprecipitated with an anti-BECN1 antibody, electrophoresed on a SDS-polyacrylamide gel and immunoblotted with and anti-BCL2 or anti-BECN1 antibody as described under Materials and Methods (upper panel). Alternatively, cellular extracts were immunoprecipitated with an anti-BLC2 and immunoblotted with an anti-BECN1 or anti-BCL2 antibody (lower panel). Densitometric analysis of the gels was performed as described under Materials and Methods. Data are representative of three separate experiments. ACTB and IgG heavy chains were used as loading controls; (**C**) MDA-MB-231 and MDA-MB-231 GLS shRNA cells were either left untreated or treated with metformin for 20 days. Where indicated in the figure, bafilomycin 100 nM was added for 17 h to the cells. At the end of the treatment cells were processed to obtain whole cellular extracts. MAP1LC3B expression was determined by Western blot. CDK4 was used as loading control. densitometric analysis of the gels was performed as described under Materials and Methods. Data are representative of three separate experiments; (**D**) left side: MDA-MB-231 cells were either left untreated or treated with metformin for 20 days (CAM cells). At the end of the treatment, medium from CAM cells was applied to fresh-seeded MDA-MB-231 cells (CCM cells) for 48 h. Both CAM and CCM cells were processed to obtain whole cellular extracts. MAP1LC3B expression was determined by Western blot. CDK4 was used as loading control. densitometric analysis of the gels was performed as described under Materials and Methods; right side: MDA-MB-231 cells were either left untreated or treated with metformin for 20 days changing medium every 2 days (fresh CAM cells). At the end of the treatment, medium from fresh CAM cells was added to fresh plated cell (fresh CCM cells). CAM cells was applied to fresh-seeded MDA-MB-231 cells (fresh CCM cells) for 48 h. Both fresh CAM and fresh CCM cells were processed to obtain whole cellular extracts. MAP1LC3B expression was determined by Western blot. CDK4 was used as loading control. Densitometric analysis of the gels was performed as described under Materials and Methods. All experiments in this figure were repeated three times.

The effects of metformin treatment on autophagic flux were evaluated by treating wt and MDA-MB-231 GLS shRNA cells with 5–30 μM metformin for 20 days. Figure 4A shows that in wt cells, metformin reduced MAP1LC3B-II, GABARAP, BECN1 and ATG12/ATG5 expression whereas, in glutaminase-silenced cells there was a reduction of only MAP1LC3B-II. However, in GLS-silenced cells, MAP1LC3-I expression was higher than in wt cells suggesting an inhibition of autophagy. Statistical analysis of blots in Figure 4A is reported in Supplementary Figure S2. In addition, we did not observe an increase in PRKAA2 phosphorylation both in wt and silenced cells (Figure 4A). This is in accordance with the observation that low doses of metformin did not alter cellular ATP production (Figure 2A). Afterwards, we checked if the interaction between BCL2 and BECN1 could be altered after metformin treatment. The BCL2/BECN1 complex represents a molecular bridge linking autophagy to apoptosis [39,40]. Therefore, we treated MDA-MB-231 cells with 30 µM metformin for 15 days and then we measured BECN1 and BCL2 binding by immunoprecipitating BECN1 and staining for BCL2 or, on the contrary, by immunoprecipitating BCL2 and staining for BECN1. In both cases, we found that metformin increased BCL2 and BECN1 binding (Figure 4B and Figure S3).

We next inhibited GLS by co-treating cells with metformin and BPTES (Figure S4A) or with metformin and dimethyl α-ketoglutarate (DMKGB) (Figure S4B). In both cases we observed an almost complete inhibition of MAP1LC3B-II. To confirm that the reduction of autophagic markers induced by metformin was due to an inhibition and not to an increase of the autophagic flux, we added bafilomycin A1 (BafA1), which inhibits autophagic vacuoles maturation causing autophagy markers accumulation [41]. As shown in Figure 4C, MAP1LC3B-II levels in MDA-MB-231 cells cotreated with 30 μM metformin and BafA1 were lower compared to BafA1 alone. Similar results were obtained in MCF7 cells (Figure S5). On the contrary, in MDA-MB-231 GLS shRNA cells we observed a weaker MAP1LC3B-II accumulation in the presence of BafA1 (Figure 4C and Figure S3).

To further prove this result, we either left untreated or treated daily MDA-MB-231 cells for 20 days with different metformin concentrations without medium replacement (CAM, cell-aged medium). At the end of treatments, we applied medium from CAM-cells to a secondary fresh-plated untreated MDA-MB-231 cultures for 48 h (CCM, cell-conditioned medium). Afterward, we monitored the autophagic markers in CAM and CCM-cells. As control we used cells where metformin containing medium was changed every two days for 20 days (fresh CAM). Again, medium from fresh CAM cells was applied to fresh seeded MDA-MB-231 cells for 48 h (fresh CCM cells). As shown in Figure 4D, untreated CAM cells presented a higher basal level of autophagy than untreated fresh CAM cells, an effect that was reduced upon metformin treatment. Interestingly, in CCM cells autophagy induction followed the trend seen in CAM cells (Figure 4D). In fact, autophagy markers were increased when using conditioned medium (CM) from control cells and decreased when using CM from metformin-treated cells (Figure 4D). Statistical analysis is reported in Supplementary Figure S3. Metformin inhibition of autophagy was MTOR independent as shown in Figure S6 where we did not observe any significant increase in RPS6KA1^Thr389^ or AKT1^Ser473^ phosphorylation neither in CAM nor in CCM cells.

**Figure 5:**
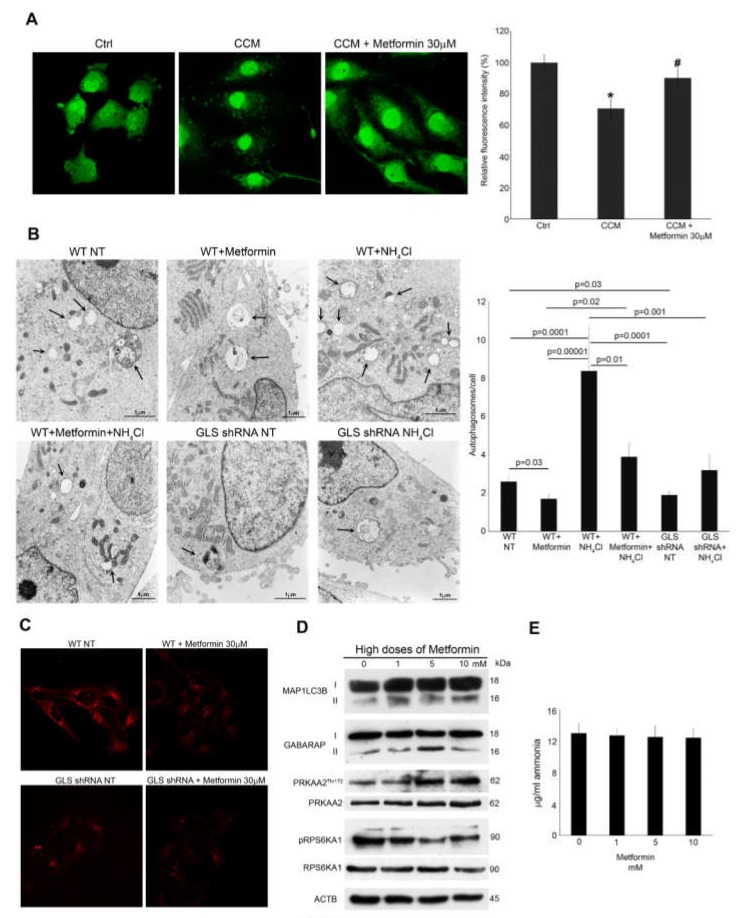
Metformin or GLS silencing reduces autophagosomes formation; (**A**) MDA-MB-231 cells (CCM) were plated in conditioned medium from CAM cells as described in Figure 4D for 48 h to stimulate autophagy in the presence or absence of metformin. At the same time MDA-MB-231 control cells (Ctrl) were plated in fresh medium for 48 h. At the end of the treatments, both Ctrl and CCM cells were labelled with AHA as described under Material and Methods. Cells were then fixed, permeabilized and stained for 2 h with alkine-Alexa Fluor 488. Fluorescence from long-lived proteins was observed using aLSM 510 confocal microscopy (Zeiss). MDA-MB-231 cells were placed in a 96 well plate, treated and labelled as described above. Right side: Fluorescence from long-lived protein was measured using a GloMax^®^-Multi Detection System (Promega). ^#^ Significantly different from CCM treated cells.; (**B**) MDA-MB-231 cells were either left untreated or treated with metformin 30 μM, NH_4_Cl 10 mM or a combination of the two. Alternatively, MDA-MB-231 GLS shRNA cells were left untreated or treated with NH_4_Cl 10 mM. The cells were then processed for electron microscopy as described under Materials and Methods. Upper left: WT cells showing autophagosomes, (Magnification 15,500×). Upper middle: WT + metformin cells showing a lower number of autophagosomes (magnification 11,500×). Upper right: WT + NH_4_Cl cells showing an increase in autophagosomes (magnification 11,500×). Lower left: WT + metformin + NH_4_Cl cells showing a reduction in autophagosomes (magnification 11,500×). Lower middle: GLS shRNA cells showing a low number of autophagosomes (magnification 15,000×). Lower right: GLS shRNA + NH_4_Cl cells showing a large autophagosome (magnification 11,500×). Black arrows point to autophagosomes and autophagolysosomes. Results were quantified on the graph reported on the right side showing reduction of autophagosomes following metformin treatment or GLS silencing. Number of autophagosomes were obtained by counting three different fields for each image from two separate experiments; (**C**) MDA-MB-231 WT and GLS shRNA cells were either left untreated or treated with metformin 30 μM for 20 days. At the end of the treatment, 50 nM Lysotracker red was added to the cells for 30 min followed by a wash in PBS before confocal analysis of lysosome staining as described under materials and methods. Upper left: WT untreated cells showing lysosomes accumulation. Upper right: reduced lysosomes in Metformin treated cells. Lower left: GLS shRNA cells showing reduced lysosome staining. Lower right: Metformin treatment did not reduce lysosomes accumulation in GLS shRNA cells; (**D**) MDA-MB-231 cells were treated with high doses of metformin from 1 to 10 mM for 48 h. At the end of the treatment, cells were processed to obtain whole cell lysates. MAP1LC3B, GABARAP, pPRKAA2^Thr172^, PRKAA2, pRPS6KA1 and RPS6KA1 expression was determined by Western blot. ACTB was used as loading control. Densitometric analysis of the gels was performed as described under Materials and Methods; (**E**) MDA-MB-231 cells were treated with high doses of metformin from 1 to 10 mM for 48 h. Ammonia level in the culture medium was measured as indicated in the Material and Methods section. All experiments in this figure were repeated three times. * Significantly different from control untreated cells.

Autophagy inhibition induced by metformin was also quantified by measuring degradation of long-lived protein through the Click-it AHA technique [42]. Again, we made use of CCM cells, that is, MDA-MB-231 cells incubated with 20-days old conditioned medium from the same cell line kept in the presence or absence of 30 μM metformin. After 2 days, CCM cells were plated in l-methionine free medium in the presence of l-azidohomoalanine (AHA). After 2 h we measured the changes of AHA fluorescence intensity, which is representative of autophagy-mediated proteolysis. We observed a decrease of AHA fluorescence in cells incubated with CCM compared to CCM plus metformin (Figure 5A, left side). Quantification of AHA fluorescence revealed a decrease of about 30% in CCM cells compared to control cells in fresh medium (Figure 5A, right side). Metformin treatment maintained AHA fluorescence to levels similar to that of Ctrl cells (Figure 5A, right side). To further confirm these data, we investigated metformin-mediated autophagy reduction by Transmission Electron Microscopy (TEM). Figure 5B shows that MDA-MB-231 cells treated with metformin had a lower number of autophagosomes than untreated cells. This reduction of autophagosomes and autophagolysosomes was clearer following ammonium chloride treatment, as evidenced in the right panels of Figure 5B. Importantly, glutaminase-silenced cells showed a reduced basal accumulation of autophagosomes and autophagolysosomes than wt cells, reproducing results obtained upon metformin treatment. These results were quantified in the graph on the right side of Figure 5B showing the reduction of autophagy due to either metformin treatment or glutaminase silencing.

Autophagy was also monitored by labelling MDA-MB-231 wt and GLS-silenced cells with lysotracker red. At first we observed a reduction of autophagy-associated lysosomes in wt cells after metformin treatment (Figure 5C, upper right). On the contrary, in GLS-silenced cells we observed a lower basal level of red fluorescence than wt cells (Figure 5C, lower left) that was not affected by metformin treatment (Figure 5C, lower right).

We also investigated the effects of high doses of metformin on MDA-MB-231 cells. In fact, when used at millimolar doses, metformin has been shown to trigger autophagy through PRKAA2/MTOR axis modulation [21]. For this reason, we analysed PRKAA2^Thr172^ phosphorylation which is representative of its activation status, the phosphorylation of RPS6KA1, which is a marker of *MTOR complex I activity (mTORC1)*, negatively regulated by PRKAA2 [43] as well as MAP1LC3B-II accumulation. Upon 48 h of metformin treatment, MAP1LC3B and PRKAA2^Thr172^ increased in a dose-dependent manner (Figure 5D). Statistical analysis of the blots in Figure 5D is reported in Supplementary Figure S7. Importantly, we did not observe a reduction of ammonia in the medium of MDA-MB-231 cells treated with millimolar concentration of metformin (Figure 5E). These findings are in line with the present literature data [44] and confirm our ATP assay results (Figure 2A). Together, these results suggest that, millimolar doses of metformin induce an energetic unbalance with ATP reduction leading to PRKAA2 activation and autophagy without altering ammonia levels.

Finally, some studies have shown that in MDA-MB-231 cells metformin is able to impair the activity of hexokinase HK1 and HK2 [45]. Therefore, we replaced high glucose medium with galactose medium before adding metformin. In this case, we treated MDA-MB-231 cells up to 10 days because the removal of glucose in association with metformin treatment strongly influenced cell proliferation, as shown in clonogenicity assay in Figure S8A. Again, we observed that metformin inhibited autophagy as evidenced by MAP1LC3B, GABARAP and ATG5/ATG12 reduction (Figure S8B) without altering PRKAA2 phosphorylation.

### 3.5. Effects of a Combined Metformin and Cisplatin Treatment

**Figure 6:**
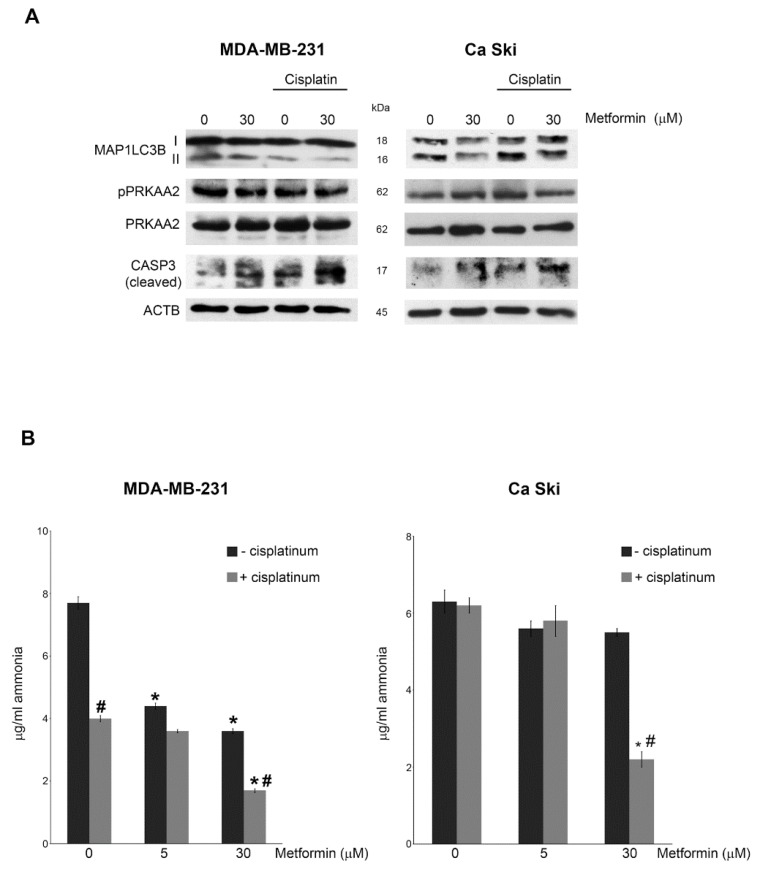
Metformin treatment increases cisplatin effects; (**A**) MDA-MB-231 and Ca Ski cells were either left untreated or treated with metformin or cisplatin alone or metformin in combination with cisplatin for 15 days. At the end of the treatment, cells were processed to obtain whole cell lysates. MAP1LC3B, pPRKAA2^Thr172^, PRKAA2 and CASP3 (cleaved) expression was determined by Western blot. ACTB was used as loading control. Densitometric analysis of the gels was performed as described under Materials and Methods; (**B**) MDA-MB-231 and Ca Ski cells were either left untreated or treated with metformin or cisplatin alone or metformin in combination with cisplatin for 15 days. Ammonia level in the culture medium was measured as indicated in the Material and Methods section. ^#^ Significantly different from treatment with metformin alone. All experiments in this figure were repeated three times. * Significantly different from control untreated cells.

Metformin is often used in gynaecological oncology as adjuvant drug to increase the efficacy of cisplatin-based neoadjuvant (NACT) chemotherapy [24], in neck and cervix cancer [46] and earlier-stage operable breast cancer [47]. For this reason, we treated cervical and breast cancer cell lines with low doses of metformin and cisplatin for 15 days. Figure 6A shows that, in MDA-MB-231 cells, the combined metformin/cisplatin treatment did not alter PRKAA2^Thr172^ phosphorylation. On the contrary MAP1LC3B-II accumulation was strongly reduced as also evidenced in Supplementary Figure S9. This trend was less relevant in Ca Ski cell line probably because breast cancer cells are more sensitive to metformin action than cervical cancer cell lines (Figure 3B). In addition, we observed that reduction of ammonia levels induced by metformin was intensified when cells were co-treated with a nontoxic concentration of cisplatin. Once again, this reduction was clearer in MDA-MB-231 cells than in Ca Ski (Figure 6B). Metformin increased the level of cleaved-CASP3 in both cell lines, an effect that was also present in cisplatin-treated cells and that was enhanced by the combined metformin and cisplatin treatment (Figure 6A). This is probably due to the reduced ammonia accumulation and inhibition of autophagy by metformin as shown in Figure 6B.

## 4. Discussion

Metformin, a biguanide commonly used for T2D therapy, can reduce the risk of cancer in diabetic patients compared to other anti-diabetic treatments [48]. Therefore, we aimed to investigate the molecular mechanism behind the anti-tumoral action of metformin. Our results demonstrate that low doses (5–30 μM) of metformin have two major effects: i) autophagy inhibition by decreasing glutamine metabolism and ammonia accumulation, ii) apoptosis induction by altering mitochondrial energization. Moreover, we also demonstrate that, along this pathway, mitochondrial GLS, involved in the first step of glutamine metabolism and often overexpressed in tumour cells, is a target of metformin.

We are aware that several groups showed that millimolar doses of metformin can inhibit Mitochondrial Complex 1, ATP production and tumour cell growth [49]. However, these in vitro concentrations are far above those measured in tissues from T2D patients assuming metformin. In fact, this drug rapidly reaches its peak (2 h) with a tissue concentration around 5–30 μM [50]. Therefore, we decided to incubate cancer cells with micromolar doses of metformin for a longer time. Surprisingly, we discovered that these doses of metformin still showed an antitumoral effect (Figure 1) increasing cell death in different tumour cell lines. Moreover, also in our hands, high doses of metformin reduce ATP levels and activate autophagy, an effect that, then, increases cell death [49]. In fact, a drop in ATP was observed when treating tumour cells with a dose of 5 mM metformin (Figure 1A) similarly to what reported using 10 mM metformin [49]. As expected this drop in ATP was accompanied by AMPK activation (Figure 5D) and autophagy induction with LC3-II and GABARAP-II accumulation (Figure 5D). By contrast, using low doses of metformin we inhibited autophagy increasing mitochondrial depolarization and apoptosis (Figure 2). At present, we do not know the precise mechanisms underneath such divergent effects of low and high metformin dosage on autophagy. We can speculate that they may be due to off-target effects obtained when using high doses, that is, above 30 μM, of metformin. Moreover, it is important to consider that these divergent effects are not a peculiarity of metformin. In fact, many anti-oxidant compounds becomes pro-oxidant if used at high concentration such as, for example, resveratrol and vitamin C [51,52] depending on the presence of transition metals or on the fact that they can be oxidized. However, in the case of metformin, it is interesting to consider that both low and high doses causes cell death by de-regulating a basic survival process such as autophagy.

However, in light of our observations, herewith, we suggest that low doses of metformin (5–30 μM) and long times of treatment (up to 20 days) may be the best in vitroconditions necessary to investigate the molecular mechanism behind the anti-tumoral effect of this drug.

A large number of cancer cells are addicted to glutamine [53] showing a high rate of glutaminolysis. Glutamine is sequentially deaminated in glutamate and then in α-ketoglutarate, an intermediate of TCA cycle. Supporting and extending the work by Ampuero et al. [17], we observed that metformin drastically reduces GLS activity in breast and cervical cancer cells without altering its cellular expression (Figure 3).

The biological relevancies of the GLS impairment induced by metformin are mainly two. On one hand, it reduces the support of α-ketoglutarate to TCA cycle leading to a deregulation of tumour cell metabolism. On the other hand, GLS inhibition reduces cellular ammonia amount. This molecule normally stimulates cellular catabolism through autophagy activation. Moreover, it can also act in a paracrine way diffusing in the intercellular space where it triggers autophagy also in neighbouring cancer cells [10]. GLS is overexpressed in cancer cells [54] and consequently ammonia levels are higher in tumours than normal tissue [55]. Our study shows that metformin can reduce cellular ammonia accumulation leading to an impairment of autophagy. In fact, when we daily added metformin to MDA-MB-231 cells for 20 days, we observed a strong reduction of some autophagic markers such as MAP1LC3B-II, GABARAP, SQSTM1 (Figure 4). Such a reduction was not due to an accelerated autophagic flux because blocking autophagosome degradation with BafA1 did not increase MAP1LC3B-II levels in metformin treated cells (Figure 4C). In fact, metformin treatment, is accompanied by the reduction of both cellular accumulation of autophagosomes and autophagic proteolysis of long-lived proteins (Figure 5A,B). to demonstrate the central role of *gls*in the autophagy impairment induced by metformin, we silenced the expression of this enzyme through shRNA. We documented that, compared to wild type cells, GLS silenced cells show reduced ammonia levels and reduced autophagy. Moreover, to confirm the involvement of the ammonia-induced autophagy in metformin action, we transferred cellular media of MDA-MB-231 cells treated for 20 days with metformin (CAM cells) to a secondary culture (CCM cells). Again, in CCM cells we observed the same autophagy reduction seen in CAM cells treated with metformin. We suppose that this is due to the reduction of ammonia accumulation impairing autophagy induction. This result is similar to the one obtained by Eng et al. [10] demonstrating that ammonia accumulating in a conditioned medium of a culture cell line induces autophagy in a secondary culture [56]. Moreover, such ammonia-induced autophagy is MTOR-independent [10]. Indeed, RPS6KA1, a marker of MTORC1 activity [57], or AKT, a marker of MTORC2 activity, [58] were not phosphorylated in CCM cells (Figure S6). Instead, in CAM cells we observed that metformin did not induce a phosphorylation of RPS6KA1 but surprisingly AKT1 seems to be progressively dephosphorylated at Ser 473. AKT1 activity is often increased in breast cancer and its activation is essential to protect cells against death insults [59]. We supposed that such AKT1 dephosphorylation is due to the activation of the apoptotic cascade that we observed after 10 days of metformin treatment (Figure 3). In fact, autophagy inhibition observed with low doses of metformin is accompanied by an increase of apoptosis. In particular, we demonstrated an increase in BECN1/BCL2 complex formation (Figure 4B) that frees BAX to bind to mitochondria membrane to induce depolarization and CYCS release (Figure 2). However, we still do not know if the effect of metformin on BECN1/BCL2 complex are direct or indirect. This is due to the fact that metformin interferes with both the apoptotic and autophagic mechanisms by impinging on mitochondria function and ammonia production, respectively. Interestingly, when we added cisplatin to metformin treatment we observed an additive decrease of ammonia accumulation and autophagy and increase of CASP3 cleavage (Figure 6A). Cisplatin is an anti-neoplastic agent used together with other drugs during the platinum-based neoadjuvant chemotherapy (NACT) to reduce gynaecological tumours [60]. However, NACT efficacy is limited by its high toxicity due to serious effects such as renal and liver dysfunctions. To this effect, metformin could be used in these patients in association with NACT therapy, to lower the dosage of anti-neoplastic drugs in NACT cocktail without altering its efficacy.

In conclusion, our results suggest that the documented anti-tumoral effect of metformin is due to its effect on GLS with inhibition of glutamine metabolism and reduction of ammonia-induced autophagy.

## Supplementary Materials

The following are available online at http://www.mdpi.com/2073-4409/8/1/49/s1, Figure S1. Densitometric analysis of Western Blots of Figure 2C. Figure S2. Densitometric analysis of Western Blots of Figure 4A. Figure S3. Densitometric analysis of Western Blots of Figure 4B–D. Figure S4. Inhibition of GLS reduces autophagy. Figure S5. Metformin inhibits autophagy in MCF7 cells. Figure S6. Autophagy inhibition by metformin is MTOR -independent. Figure S7. Densitometric analysis of Western Blots of Figure 5D. Figure S8. Metformin inhibits autophagy in galactose supplemented medium. Figure S9. Densitometric analysis of Western Blots of Figure 6A.

## Abbreviations

ACTB	Actin, b
AHA	l-azidohomoalanine
AKT1	Thymoma viral proto-oncogene homolog 1
ATG	Autophagy-related
BAX	BCL2-associated X protein
BCL2	B-cell CLL/lymphoma 2
BECN1	Beclin 1
BPTES	*Bis*-2-(5-phenylacetamido-1, 3, 4-thiadiazol-2-yl)ethylsulfide
CAM	Cell-aged medium
CASP3	Caspase 3
CCM	Cell-conditioned medium
CDK4	Cyclin dependent kinase 4
CYCS	Cytochrome c
GABARAP	GABA(A) receptor-associated protein
GLS	Glutaminase
HK	Hexokinase
MAP1LC3B	Microtubule-associated protein 1 light chain 3 b
MAPK	Mitogen-activated protein kinases
IGF	Insulin growth factor
JC-1	5,5′,6,6′-tetrachloro-1,1′3,3′-tetrathylbenzimidazolyl-carbocyanine iodide
mTOR	Mechanistic target of rapamycin
mTORC	mTOR complex
PHB	Prohibitin
PI3K	Phosphatidylinositol 3-kinase;
NACT	Neoadjuvant chemotherapy
PRKAA2	Protein kinase AMP-activated
RPS6KA1	Ribosomal protein S6 kinase A1
SQSTM1	Sequestosome 1
T2D	Type 2 diabetes
TCA	Tricarboxylic acid cycle
TEM	Transmission electron microscopy
